# Congratulatory Message

**DOI:** 10.31662/jmaj.s0004

**Published:** 2018-09-28

**Authors:** Barbara L. McAneny

**Affiliations:** 1 American Medical Association

Dear Colleagues,

The American Medical Association (AMA) congratulates the Japan Medical Association (JMA) on the debut of its new academic publication, *JMA Journal*, which continues JMA’s proud tradition of scientific discovery and research in international medicine.

We at the AMA have great respect for how scientific study advances clinical care, which is why we are continually improving and expanding the reach of our own *Journal of the American Medical Association*, now in its 135^th^ year, to meet the ever-changing needs of physicians, researchers and scientists.

One of the greatest challenges we as physicians face in health care today is ensuring high medical standards and elevating the quality of care delivered to patients across the globe. *JMA Journal* is another essential resource in these efforts, promoting groundbreaking research, ideas and innovative approaches to improve care delivery and inform changes in health policy.

The AMA and JMA each represent the voices of our nations’ physicians and we have a shared goal of inspiring new physician leaders, galvanizing support around issues important to the medical community, and promoting the highest ethical standards in medicine. Now, more than ever, organizations like ours have a responsibility to work collaboratively to improve health outcomes for patients and reduce health disparities in our home countries.

The AMA applauds JMA’s continued pursuit of that mission and, with you, celebrates this important milestone that advances our profession and supports improved health at home and abroad.

Sincerely,

Barbara L. McAneny, MD

President, American Medical Association

